# Integrative analysis of key candidate genes and signaling pathways in ovarian cancer by bioinformatics

**DOI:** 10.1186/s13048-021-00837-6

**Published:** 2021-07-12

**Authors:** Cuicui Dong, Xin Tian, Fucheng He, Jiayi Zhang, Xiaojian Cui, Qin He, Ping Si, Yongming Shen

**Affiliations:** 1 Department of Clinical Lab, The Children’s Hospital of Tianjin (Children’s Hospital of Tianjin University), No. 238, Longyan Road, Beichen District Tianjin, 300000 PR China; 2 Department of Medical Laboratory, The First Affiliated Hospital of Zhengzhou University, Zhengzhou, Henan Province China

**Keywords:** Ovarian Cancer, Gene Expression Omnibus, Bioinformatics Analysis, Hub Genes

## Abstract

**Background:**

Ovarian cancer is one of the most common gynecological tumors, and among gynecological tumors, its incidence and mortality rates are fairly high. However, the pathogenesis of ovarian cancer is not clear. The present study aimed to investigate the differentially expressed genes and signaling pathways associated with ovarian cancer by bioinformatics analysis.

**Methods:**

The data from three mRNA expression profiling microarrays (GSE14407, GSE29450, and GSE54388) were obtained from the Gene Expression Omnibus (GEO) database. Differentially expressed genes between ovarian cancer tissues and normal tissues were identified using R software. The overlapping genes from the three GEO datasets were identified, and profound analysis was performed. The overlapping genes were used for pathway and Gene Ontology (GO) functional enrichment analysis using the Metascape online tool. Protein–protein interactions were analyzed with the Search Tool for the Retrieval of Interacting Genes/Proteins (STRING). Subnetwork models were selected using the plugin molecular complex detection (MCODE) application in Cytoscape. Kaplan–Meier curves were used to analyze the univariate survival outcomes of the hub genes. The Human Protein Atlas (HPA) database and Gene Expression Profiling Interactive Analysis (GEPIA) were used to validate hub genes.

**Results:**

In total, 708 overlapping genes were identified through analyses of the three microarray datasets (GSE14407, GSE29450, and GSE54388). These genes mainly participated in mitotic sister chromatid segregation, regulation of chromosome segregation and regulation of the cell cycle process. High CCNA2 expression was associated with poor overall survival (OS) and tumor stage. The expression of CDK1, CDC20, CCNB1, BUB1B, CCNA2, KIF11, CDCA8, KIF2C, NDC80 and TOP2A was increased in ovarian cancer tissues compared with normal tissues according to the Oncomine database. Higher expression levels of these seven candidate genes in ovarian cancer tissues compared with normal tissues were observed by GEPIA. The protein expression levels of CCNA2, CCNB1, CDC20, CDCA8, CDK1, KIF11 and TOP2A were high in ovarian cancer tissues, which was further confirmed via the HPA database.

**Conclusion:**

Taken together, our study provided evidence concerning the altered expression of genes in ovarian cancer tissues compared with normal tissues. In vivo and in vitro experiments are required to verify the results of the present study.

**Supplementary Information:**

The online version contains supplementary material available at 10.1186/s13048-021-00837-6.

## Introduction

Ovarian cancer is one of the most common gynecological tumors associated with poor survival in women worldwide [1, 2]. The majority of newly diagnosed ovarian cancer patients are treated with radical surgery, followed by six to eight cycles of adjuvant platinum and taxane combination chemotherapy [3]. Three or more cycles of neoadjuvant chemotherapy prior to debulking surgery and adjuvant chemotherapy are alternative options for some patients [3]. However, the 5-year survival rate of ovarian cancer patients is approximately 30% [4]. Therefore, it is crucial to understand the pathogenesis of ovarian cancer. Next-generation sequencing (NGS) technology has become widely available and is used to determine a patient's precise genetic profile and to identify novel mutations to serve as new drug targets [5]. Furthermore, NGS-based mutation panels profile multiple genes simultaneously, allowing for the reporting of numerous genes while saving labor and resources [5]. Gene expression microarrays have been used in many studies to identify differentially expressed genes (DEGs) and pathways associated with ovarian cancer.

A comprehensive analysis of the interactions between DEGs and enriched pathways will contribute to the understanding of the physiopathology of ovarian cancer progression and tumorigenesis. With the increase in the number of publications about GEO datasets, GEO has become a potential resource for bioinformatics studies.

In this study, we downloaded three datasets (GSE14407, GSE29450, and GSE54388) from the Gene Expression Omnibus (GEO) (http://www.ncbi.nlm.nih.gov/geo) database. Furthermore, R software with the limma package was used to identify DEGs.

Moreover, a Venn diagram was generated to identify DEGs between ovarian cancer tissues and normal tissues that overlapped in the three GEO profiles. Subsequently, we conducted functional enrichment analyses and established a protein–protein interaction (PPI) network. Kaplan–Meier plotter, the Human Protein Atlas (HPA) database, the Oncomine database and Gene Expression Profiling Interactive Analysis (GEPIA) were used to validate the selected hub genes. In conclusion, the current research based on bioinformatics was designed to explore some potential molecular biomarkers of ovarian cancer.

## Material and methods

Ethics committee or institutional review board approval was not needed for this study, as all the data originated from a public database.

### Differentially expressed genes analysis

Three gene expression profiles (GSE54388, GSE14407, and GSE29450) were downloaded from the GEO database. GSE54388 contains 6 human ovarian surface epithelium samples and 16 tumor epithelial component samples. GSE14407 contains 12 healthy ovarian surface epithelium samples and 12 serous ovarian cancer epithelium samples. GSE29450 contains 10 ovarian cancer cell specimens and 10 normal ovarian surface epithelium specimens. The GSE54388, GSE14407, and GSE29450 data were all obtained with the Affymetrix Human Genome U133 Plus 2.0 Array platform. Expression data were normalized using median normalization [6]. DEGs between ovarian carcinoma tissues and normal tissues were obtained by using R statistical software and the limma Bioconductor package [7]. The criteria for DEG screening were FDR adjusted P < 0.05 and |logFC|≥ 1. Volcano plots and heatmaps were generated with R (Bioconductor, Roswell Park Cancer Institute, Buffalo NY, USA).

### Functional enrichment analyses of overlapping DEGs

We used the online software Venn diagram (http://www.bioinformatics.com.cn/static/others/jvenn/example.html) to identify the overlapping genes in the 3 GEO datasets [8]. These common genes were submitted to the Metascape online tool [9] and used for Gene Ontology (GO) and Kyoto Encyclopedia of Genes and Genomes (KEGG) analyses. Thresholds were set to *P-*value < 0.05, minimum count of 3, and enrichment factor of > 1.5. The top 20 GO and KEGG terms were selected and are shown in the graphs.

### PPI network

These common genes were submitted to the Search Tool for the Retrieval of Interacting Genes (STRING; http://string-db.org) (version 11.0) and visualized using Cytoscape. The minimum required interaction score was set to 0.9, and disconnected nodes in the network were hidden. Then, we used the MCODE app of Cytoscape to indentify the significant modules in the PPI network (maximum depth = 100, node score cutoff = 0.2, degree cutoff = 2, and k-core = 2) [10]. The top five MCODE models are listed, and the top ten hub genes were used for further analyses.

### Validation of the hub genes

The expression levels of the selected hub genes was further analyzed using the Oncomine database (http://www.oncomine.org) and GEPIA database (http://gepia.cancer-pku.cn/). Boxplots, stage plots and survival analyses for these hub genes were further performed using the GEPIA database (http://gepia.cancer-pku.cn/).

Moreover, the HPA database (http://www.proteinatlas.org/) was searched and retrieved to compare the protein expression of hub genes in cancerous specimens with that in normal specimens. The HPA database, comprising more than 10 million pictures showing human protein expression patterns in tissues and cells, is publicly available to allow researchers to freely study the human proteome [11].

## Results

### Identification of DEGs in ovarian cancers

**Fig. 1:**
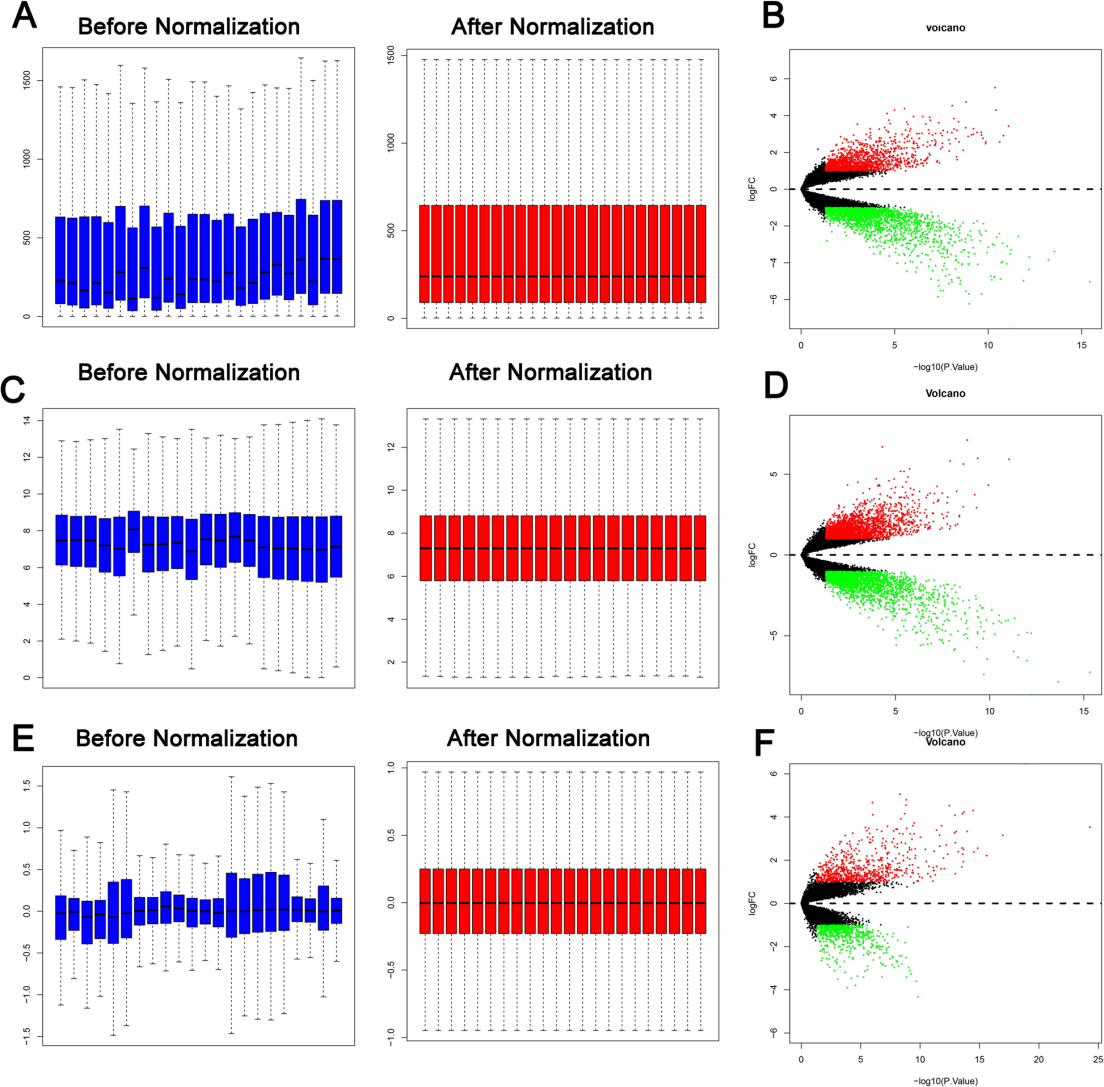
Differentially expressed genes in GSE54388, GSE14407, and GSE29450. **A** Box plot comparing the distribution of the expression values for all the samples after normalization in GSE54388. **B** Volcano plot of differentially expressed genes (DEGs) in GSE29450. Green dots represent downregulated genes and red dots represent upregulated genes in breast cancer tissue; black dots represent normally expressed genes. **C** Box plot comparing the distribution of the expression values for all the samples after normalization in GSE14407. **D** Volcano plot of differentially expressed genes (DEGs) in GSE14407. Green dots represent downregulated genes and red dots represent upregulated genes in breast cancer tissue; black dots represent normally expressed genes. **E** Box plot comparing the distribution of the expression values for all the samples after normalization in GSE29450. **F** Volcano plot of differentially expressed genes (DEGs) in GSE29450. Green dots represent downregulated genes and red dots represent upregulated genes in breast cancer tissue; black dots represent normally expressed genes

**Fig. 2:**
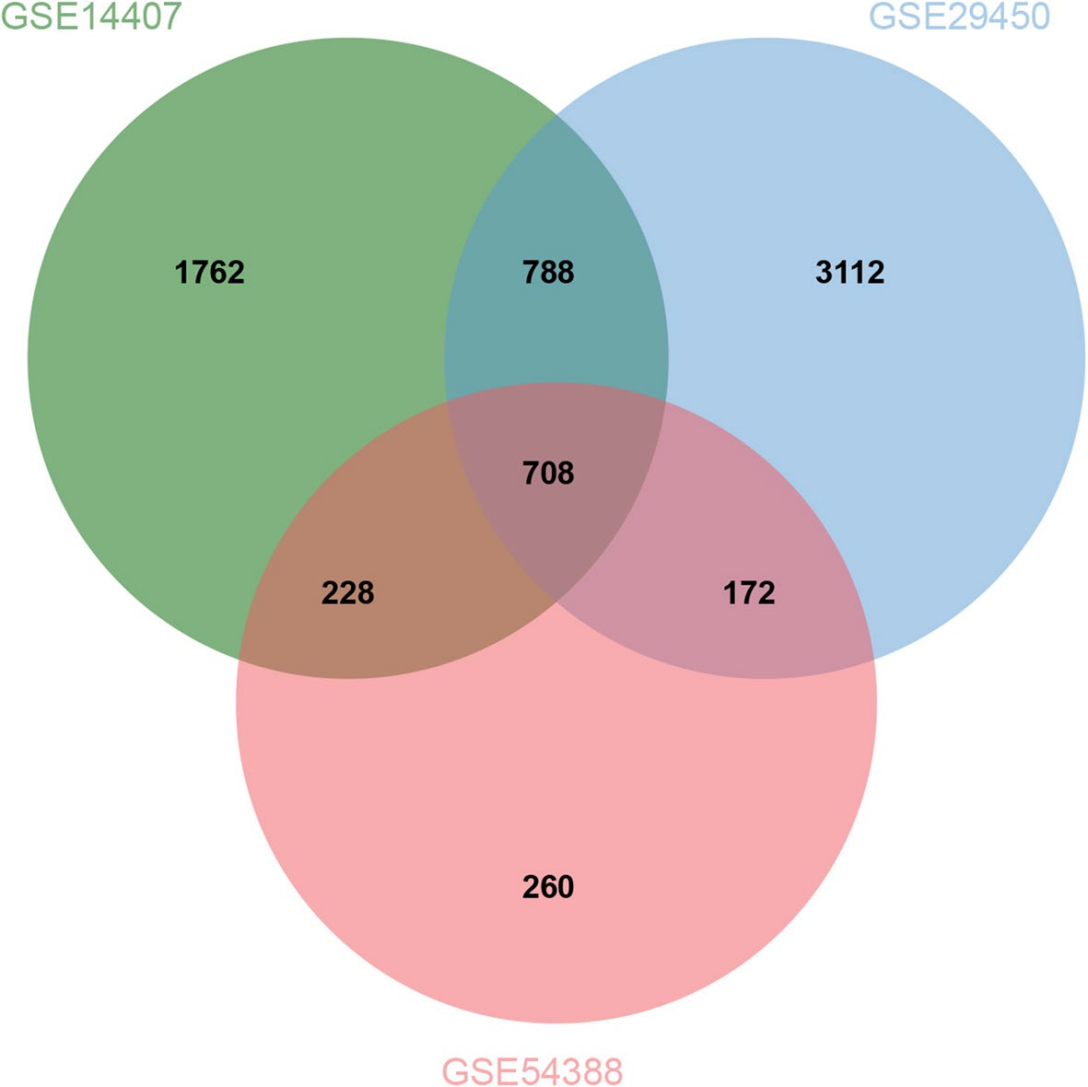
Venn diagram presenting the number of overlapping genes among three GEO datasets (GSE54388, GSE14407, and GSE29450)

First, we normalized the read counts for each sample. The median values of each sample were almost consistent, suggesting that the data were eligible for further analysis (Fig. 1 A, C and E). A total of 3486 DEGs (upregulated = 1950; downregulated = 1536) were identified in GSE14407 (Fig. 1 B), and the top ten DEGs are listed in Table 1. A total of 4780 DEGs (upregulated = 2407; downregulated = 2373) were identified in GSE29450 (Fig. 1 D), and the top ten DEGs are listed in Table 2. A total of 1368 DEGs (upregulated = 625; downregulated = 743) were identified in GSE54388 (Fig. 1 F), and the top ten DEGs are listed in Table 3. Then, 708 overlapping genes were finally selected for further analysis (Fig. 2).

**Table 1:** Top ten differentially expressed genes in ovarian cancer than normal tissue in GSE14407

Gene	logFC	AveExpr	t	*P* Value	adj.P.Val	B
NELL2	-5.0322	11.33906	-17.8843	3.62E-16	7.84E-12	26.13203
TXNIP	-3.38158	11.92188	-14.8911	2.85E-14	3.09E-10	22.23984
ARHGAP18	-3.83976	11.43925	-14.4286	5.96E-14	4.30E-10	21.56643
RERG	-3.69045	9.294558	-13.0174	6.38E-13	2.85E-09	19.37512
AQP9	-4.36576	9.957475	-12.9985	6.59E-13	2.85E-09	19.34429
REEP1	-4.97147	9.464285	-12.6144	1.30E-12	4.69E-09	18.70929
FRY	-2.55897	8.842311	-12.2756	2.40E-12	7.41E-09	18.13482
ANKRD29	-3.29846	8.646326	-11.7771	6.03E-12	1.58E-08	17.26458
DPYD	-3.52501	9.751954	-11.7269	6.62E-12	1.58E-08	17.17536
ITLN1	-7.58571	10.52319	-11.6755	7.30E-12	1.58E-08	17.0836

**Table 2:** Top ten differentially expressed genes in ovarian cancer than normal tissue in GSE29450

Gene	logFC	AveExpr	t	*P* Value	adj.P.Val	B
BNC1	-7.25722	8.828002	-21.0627	4.69E-16	1.02E-11	25.517
CALB2	-7.84629	10.12555	-17.457	2.32E-14	2.51E-10	22.25684
ITLN1	-8.65669	11.21177	-14.8908	5.86E-13	3.38E-09	19.40216
LINC01105	-4.83459	7.945648	-14.8442	6.24E-13	3.38E-09	19.34546
LINC00842	-4.7977	5.78064	-14.489	1.01E-12	3.88E-09	18.90674
CLEC4M	-6.52877	8.317774	-14.4461	1.07E-12	3.88E-09	18.85291
C21orf62	-4.94547	7.809939	-13.7446	2.89E-12	8.39E-09	17.94981
GPR133	-6.31894	7.815952	-13.6959	3.10E-12	8.39E-09	17.88546
PEX5L	-3.90997	6.84449	-13.4133	4.67E-12	1.12E-08	17.50705
SERTM1	-5.97807	9.900058	-13.3287	5.29E-12	1.15E-08	17.39221

**Table 3:** Top ten differentially expressed genes in ovarian cancer than normal tissue in GSE54388

Gene	logFC	AveExpr	t	*P* Value	adj.P.Val	B
LINC01105	3.533815	0.858675	50.96816	4.99E-25	1.08E-20	40.50396
ITLN1	6.462358	1.614636	29.20985	1.37E-19	1.48E-15	32.75399
WNT2B	3.157712	0.658476	23.96666	1.09E-17	7.89E-14	29.30784
ABCA8	2.207886	0.566936	20.76557	2.51E-16	1.36E-12	26.65956
ADH1C	2.551191	0.469443	19.17573	1.41E-15	6.11E-12	25.14902
CLDN15	2.384036	0.47813	18.51044	3.01E-15	1.07E-11	24.47264
MGARP	4.30019	1.069266	18.39105	3.46E-15	1.07E-11	24.34831
CALB2	3.326602	0.818659	17.43105	1.09E-14	2.95E-11	23.31443
PRG4	4.229521	1.027993	16.90125	2.10E-14	4.27E-11	22.71661
LHX2	2.50154	0.618326	16.86466	2.20E-14	4.27E-11	22.67457

### KEGG and GO enrichment analyses

**Fig. 3:**
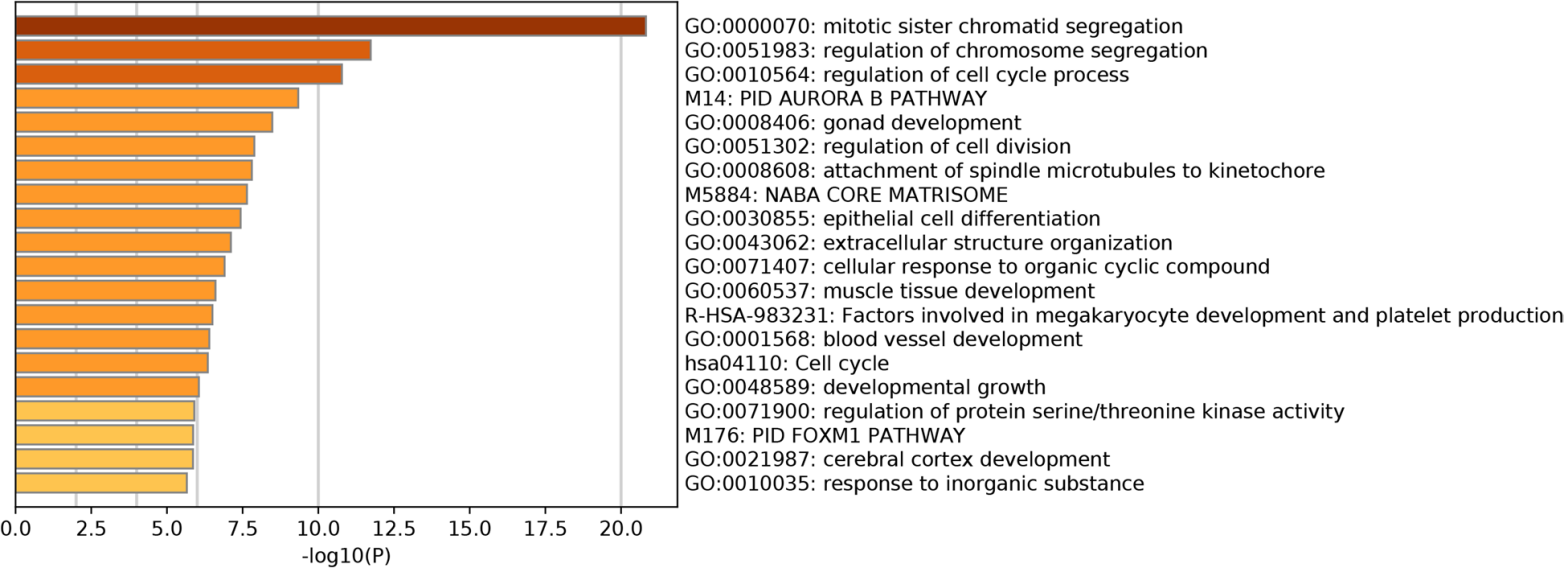
Top 20 GO and KEGG enriched terms of overlapping genes. Different colors represent different enrichments; the darker the color is, the higher the enrichment is. The horizontal axis represents the -log10 *P-*value, and the vertical axis represents the enriched terms

Next, we identified enriched gene ontology and KEGG pathway annotations associated with the overlapping genes. The top twenty GO and KEGG terms were as follows (Fig. 3): mitotic sister chromatid segregation, regulation of chromosome segregation, regulation of cell cycle process, PID AURORA B Pathway, gonad development, regulation of cell division, attachment of spindle microtubules to kinetochore, NABA CORE MATRISOME, epithelial cell differentiation, extracellular structure organization, cellular response to organic cyclic compound, muscle tissue development, factors involved in megakaryocyte development and platelet production, blood vessel development, cell cycle, development growth, regulation of protein serine/threonine kinase activity, PID FOXM1 PATHWAY, cerebral cortex development, and response to inorganic substance.

### PPI network and significant module analysis

**Fig. 4:**
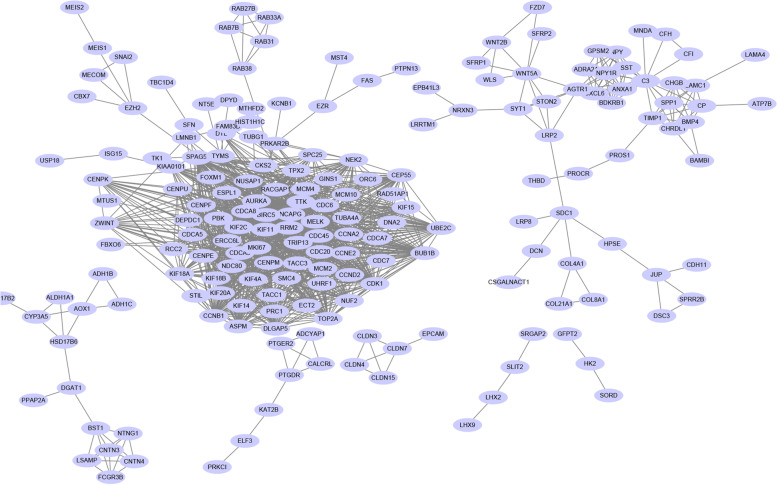
PPI network of overlapping genes. Solid lines indicate a direct connection

**Fig. 5:**
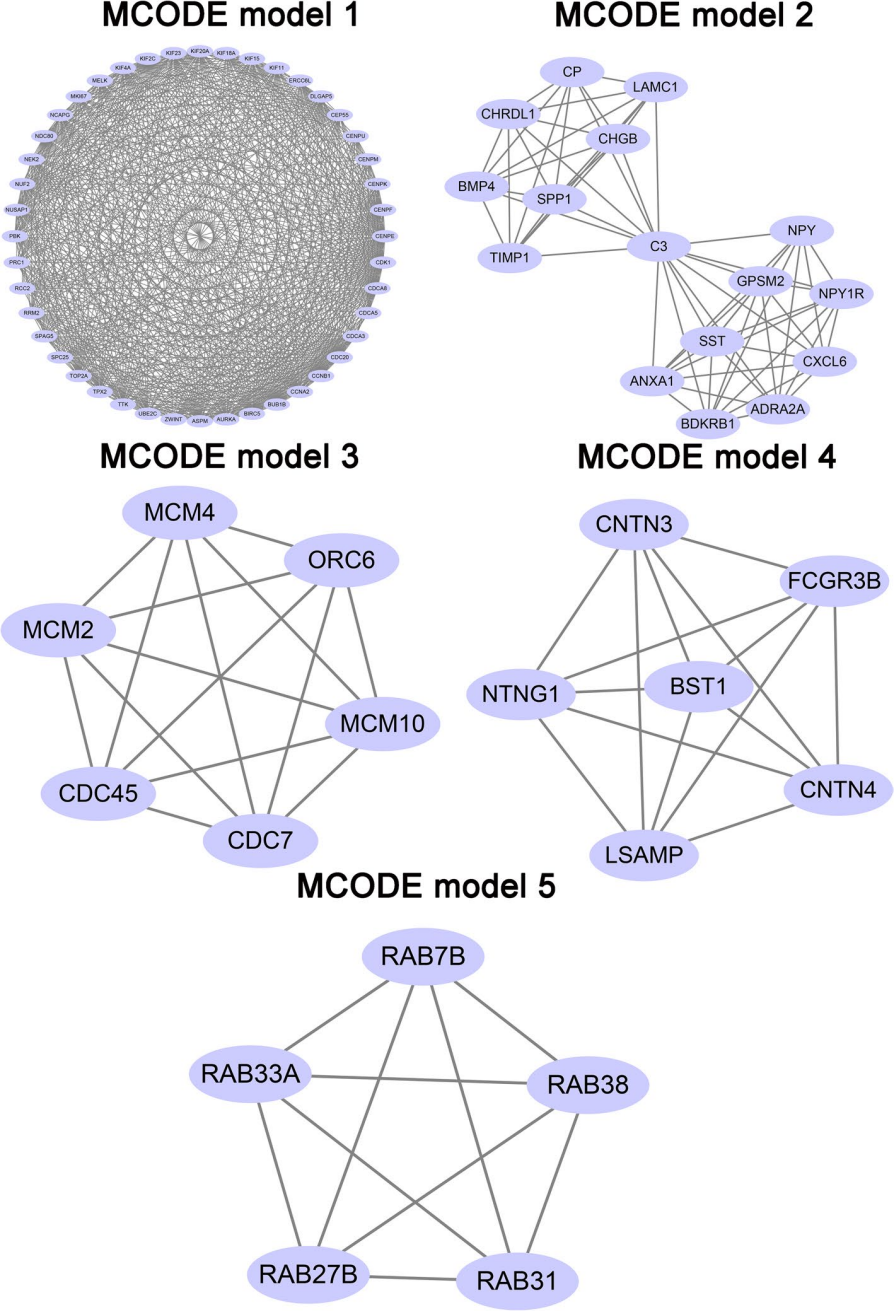
Top 5 primary modules of the PPI subnetwork by plug-in MCODE in Cytoscape software

We set the minimum required interaction score to 0.09 in the STRING database. There were 656 nodes and 1231 edges in this PPI network (Fig. 4). The most significant module, identified using the MCODE app in Cytoscape, is shown in Fig. 5.

**Fig. 6:**
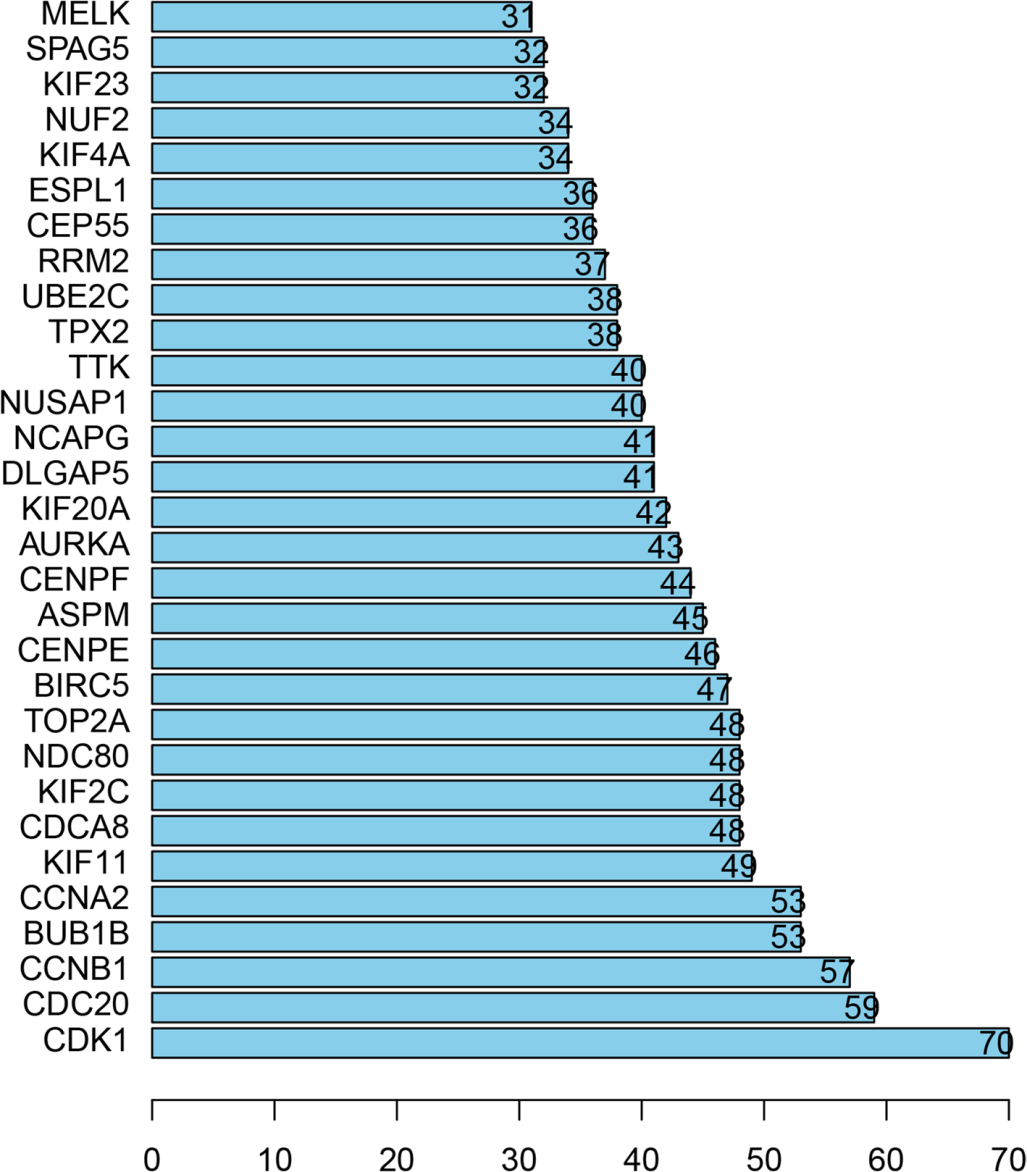
The top 30 core proteins of the PPI network. The ordinate coordinates represent the name of the gene, and the abscissa represents the number of gene connections

We selected the top 10 hub genes (CDK1, CDC20, CCNB1, BUB1B, CCNA2, KIF11, CDCA8, KIF2C, NDC80 and TOP2A, Fig. 6) according to the joint points from the STRING database.

### Validation of the hub genes

**Fig. 7:**
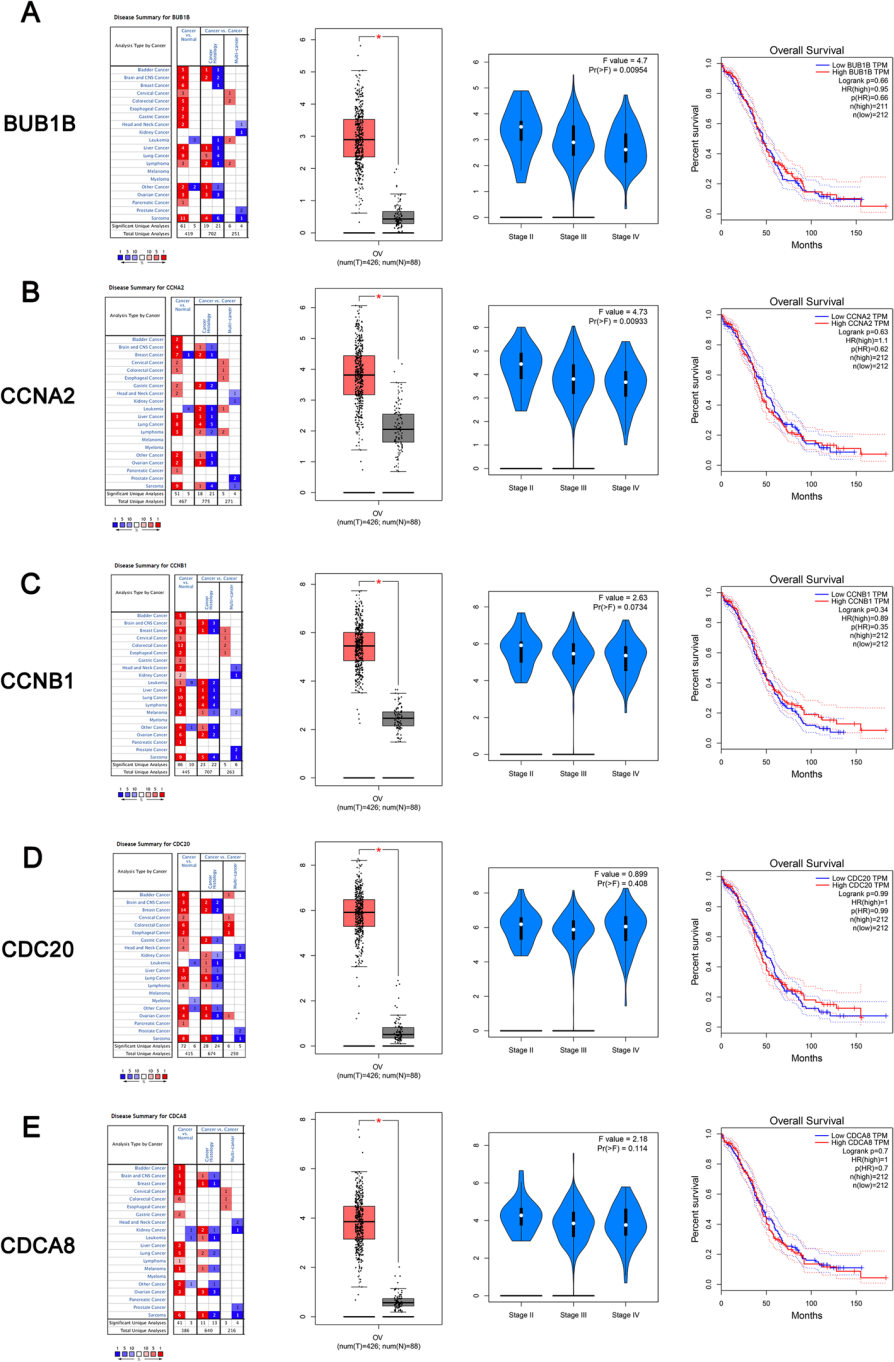
**A** BUB1B expression and overall survival analyses in ovarian cancer tissues compared with normal tissues in the Oncomine database and GEPIA database, **B** CCNA2 expression and overall survival analyses in ovarian cancer tissues compared with normal tissues in the Oncomine database and GEPIA database. **C** CCNB1 expression and overall survival analyses in ovarian cancer tissues compared with normal tissues in the Oncomine database and GEPIA database. **D** CDC20 expression and overall survival analyses in ovarian cancer tissues compared with normal tissues in the Oncomine database and GEPIA database; **E** CDCA8 expression and overall survival analyses in ovarian cancer tissues compared with normal tissues in the Oncomine database and GEPIA database

**Fig. 8:**
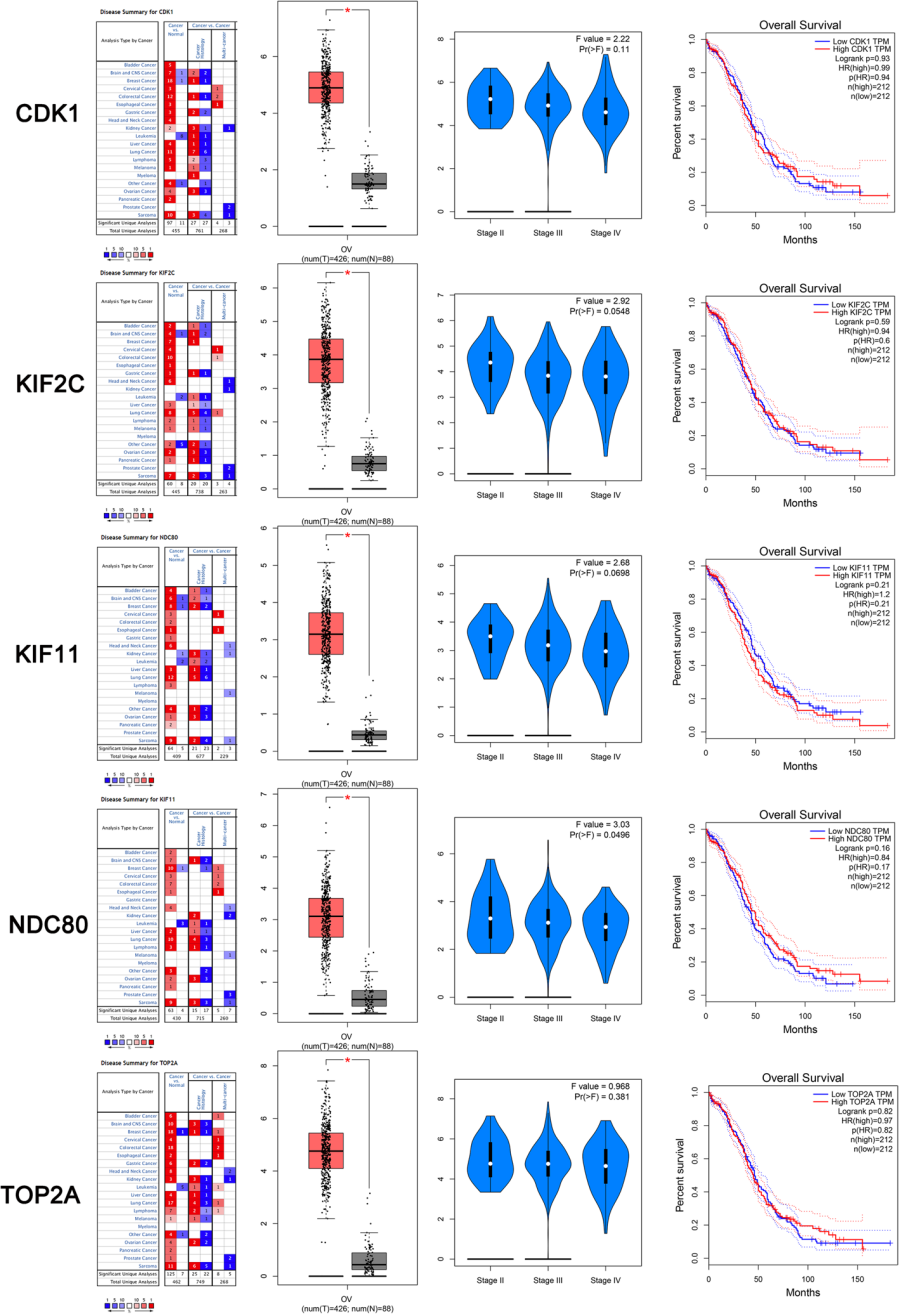
CDK1 expression and overall survival analyses in ovarian cancer tissues compared with normal tissues in the Oncomine database and GEPIA database, **B** KIF2C expression and overall survival analyses in ovarian cancer tissues compared with normal tissues in the Oncomine database and GEPIA database. **C** KIF11 expression and overall survival analyses in ovarian cancer tissues compared with normal tissues in the Oncomine database and GEPIA database. **D** NDDC80 expression and overall survival analyses in ovarian cancer tissues compared with normal tissues in the Oncomine database and GEPIA database; **E** TOP2A expression and overall survival analyses in ovarian cancer tissues compared with normal tissues in the Oncomine database and GEPIA database

To determine differences in the expression of these ten hub genes in ovarian cancer tissues and normal tissues, the hub gene mRNA levels in ovarian cancer tissues and normal tissues were further analyzed using the Oncomine database. The results revealed that CDK1, CDC20, CCNB1, BUB1B, CCNA2, KIF11, CDCA8, KIF2C, NDC80 and TOP2A were all expressed at higher levels in ovarian cancer tissues than in normal tissues (Fig. 7 and 8).

Correlations between CDK1, CDC20, CCNB1, BUB1B, CCNA2, KIF11, CDCA8, KIF2C, NDC80 and TOP2A expression and tumor stage were further analyzed in ovarian cancer patients (GEPIA). The distribution of BUB1B and CCNA2 expression correlated with tumor stage.

**Fig. 9:**
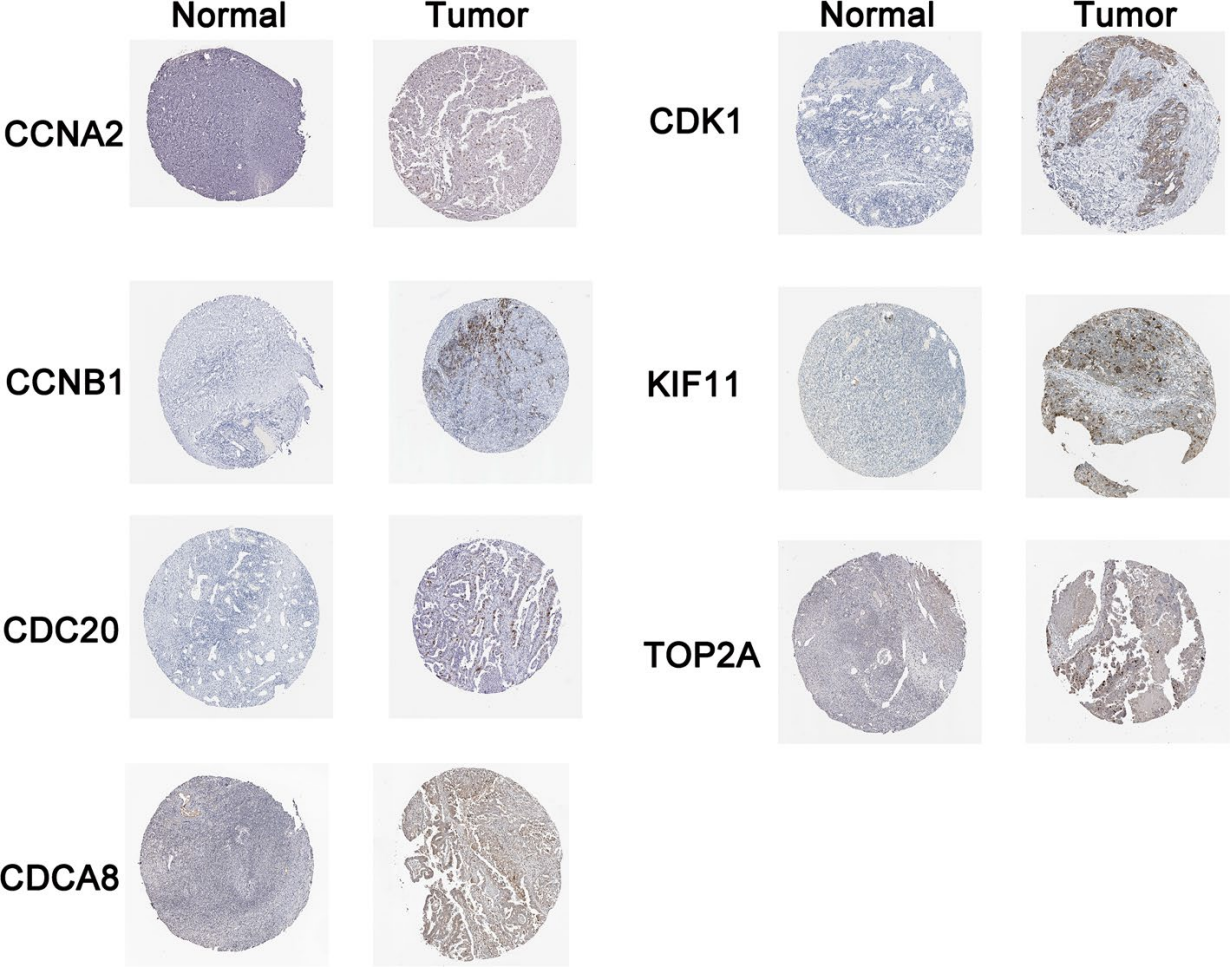
Validation of the total 7 DEGs from the HPA database by immunohistochemistry

The high protein expression levels of CCNA2 in the cancerous samples were further confirmed by the results from the HPA dataset (Fig. 9). The KIF2C and BUB1B protein data were missing from the HPA dataset. Finally, we identified CCNA2 as the hub gene that is crucial for ovarian cancer progression.

## Discussion

The present study is the first to explore DEGs between ovarian cancer tissues and normal tissues with three GEO datasets. We finally determined that CCNA2 that was more highly expressed in ovarian cancer, and an increased CCNA2 RNA expression level was associated with poor PPS in all patients with ovarian cancer. These findings may contribute to the understanding of the pathogenesis of ovarian cancer and thus aid in the improvement of diagnosis, treatment and patient outcome.

To identify DEGs between ovarian cancer tissues and normal tissues, we first analyzed three GEO datasets and identified 708 overlapping genes. Then, the Metascape online tool was used to further confirm the function of these genes. These DEGs were mainly enriched in the regulation of cell cycle process, cell division and blood vessel development. Finally, we identified ten hub genes (CDK1, CDC20, CCNB1, BUB1B, CCNA2, KIF11, CDCA8, KIF2C, NDC80 and TOP2A) for further analysis. Overexpression of CDK1, CCNB1 and CDC20 in tumor tissues predicted poor survival of patients with hepatocellular carcinoma [12]. The results of this study suggest that these genes are also involved in regulating the tumorigenesis and progression of hepatocellular carcinoma. However, the survival rate associated with the high expression and low expression of these 3 genes, as assessed via the Kaplan–Meier method, was not significantly different.

According to Oncomine and GEPIA, these ten hub genes were more highly expressed in ovarian cancer tissues than in normal tissues. Finally, we identified CCNA2 and BUB1B as candidate hub genes for ovarian cancer progression, as their high expression was correlated with tumor stage and overall survival of ovarian cancer.

CCNA2 protein expression was further confirmed by the HPA database, which suggested that CCNA2 has potential prognostic and therapeutic significance in ovarian cancer. CCNA2 upregulation is also reportedly associated with the progression of other malignancies, including gastric cancer [13], hepatocellular carcinoma [14] and lung squamous cell carcinoma [15]. CCNA2 is a key regulatory protein that promotes the transition from S phase to G2/M phase [16].

Gene ontology analysis revealed that CCNA2 was mainly enriched in mitotic sister chromatid segregation, cell division and the cell cycle. Mitotic DNA repair is thought to primarily involve sister chromatids. Abnormal mitotic DNA repair is closely related to tumorigenesis and progression [17].

The repair of DNA damage is crucial for the maintenance of genomic integrity. Cells cannot properly repair their DNA during replication without the complete set of DNA repair proteins at a damage site. In these circumstances, the outcome is mitotic catastrophe and subsequent cell death. The proteins encoded by the breast cancer gene 1/2 (BRCA1/2) genes participate in the repair of DNA double strand breaks. The loss of function of these genes renders cancer cells more dependent on alternative DNA repair mechanisms, such as single-strand DNA repair. Poly(ADP-ribose) polymerase (PARP) inhibition in breast cancer mutant tumor cells induces synthetic lethality and has emerged as a promising anticancer therapy, especially in BRCA1/2 mutation carriers [18].

In tumorigenesis, the apoptotic cell division ratio is altered [19]. Cancer cells undergo uncontrolled cell division without programmed cell death or apoptosis [20]. Dysregulation of cell cycle progression is also considered a common characteristic of cancer [21]. CCNA2 is involved in biological processes of the cell cycle. Therefore, studies with a larger number of samples will be used to verify the results of the present study in the future.

The BUB1B gene is located at chromosome 15q15 and plays a vital role in chromosome segregation [22]. Ding et al. [23] revealed that BUB1B, CDK1, CCNA2, TOP2A, BUB1B, and KIF11 were hub genes in the progression of colorectal cancer, and these genes were all differentially expressed in ovarian cancer. Similar findings were reported by two other research groups and showed that BUB1B participates in tumor growth and the progression of prostate cancer and lung adenocarcinoma [24, 25]. A previous study performed KEGG enrichment analysis and indicated that BUB1B was associated with the cell cycle [26]. Halting cell cycle progression is crucial for the development of tumorigenesis and tumor progression [27].

This study has some limitation. First, the pathological data were incomplete and were not included in the results of this study. Second, there was no experimental verification.

## Conclusion

Taken together, our study provided evidence concerning the altered expression of genes in ovarian cancer tissues compared with normal tissues. In vivo and in vitro experiments are required to verify the results of the present study.

## Supplementary Information

**Additional file 1: Supplement S1** Heatmap for GSE14407, GSE29450, and GSE54388.

## Abbreviations

GEO: Gene Expression Omnibus
GO: Gene Ontology
STRING: Search Tool for the Retrieval of Interacting Genes/Proteins
MCODE: Molecular complex detection
HPA: Human Protein Atlas
GEPIA: Gene Expression Profiling Interactive Analysis
OS: Overall survival
DEGs: Differentially expressed genes
PPI: Protein–protein interaction
KEGG: Kyoto Encyclopedia of Genes and Genomes
