# Supplementary figures and images for: Integrative analysis of key candidate genes and signaling pathways in ovarian cancer by bioinformatics
Source: J Ovarian Res. 2021 Jul 12;14:92. doi: 10.1186/s13048-021-00837-6 (PMC8276467; doi:10.1186/s13048-021-00837-6)

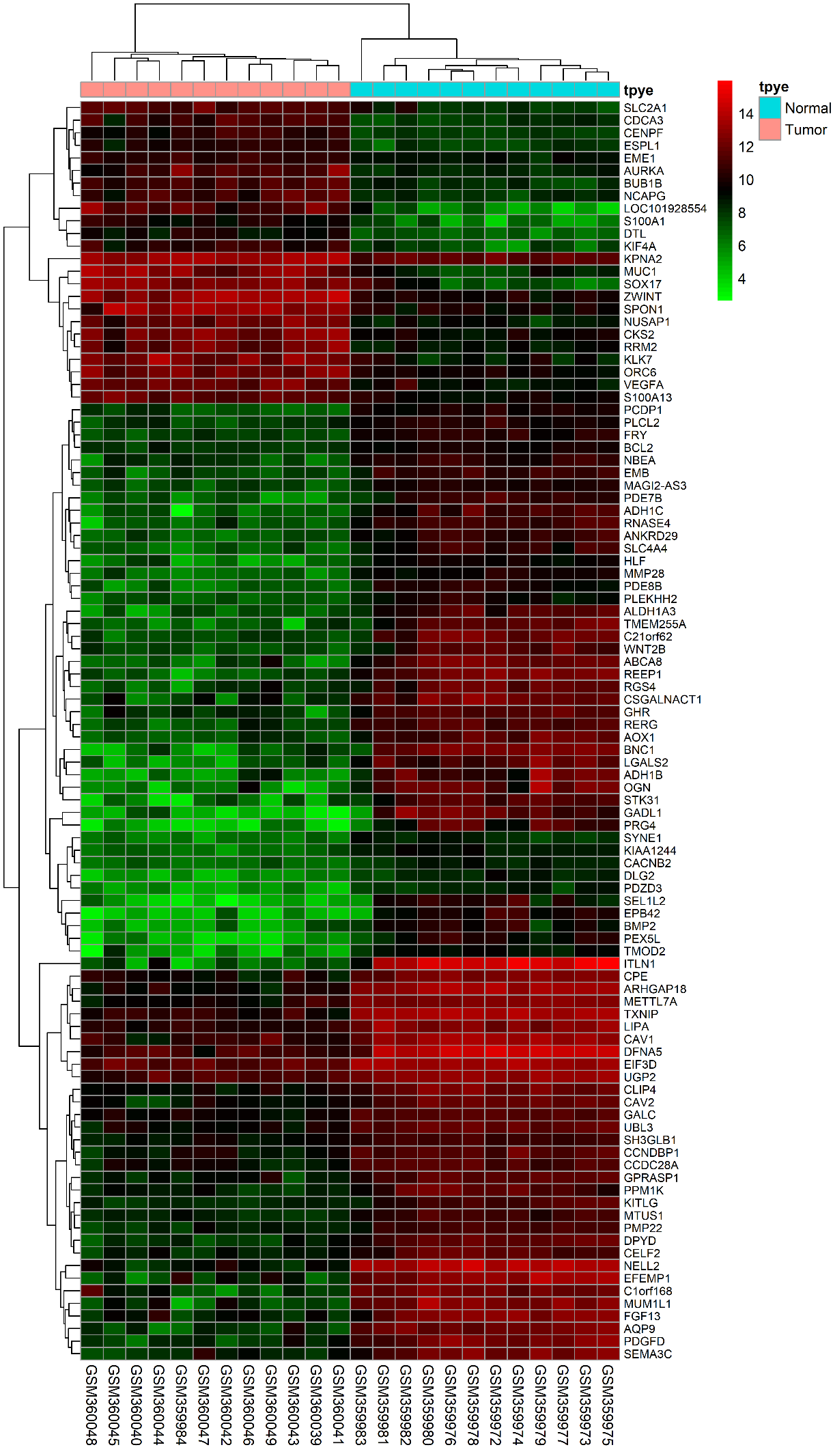


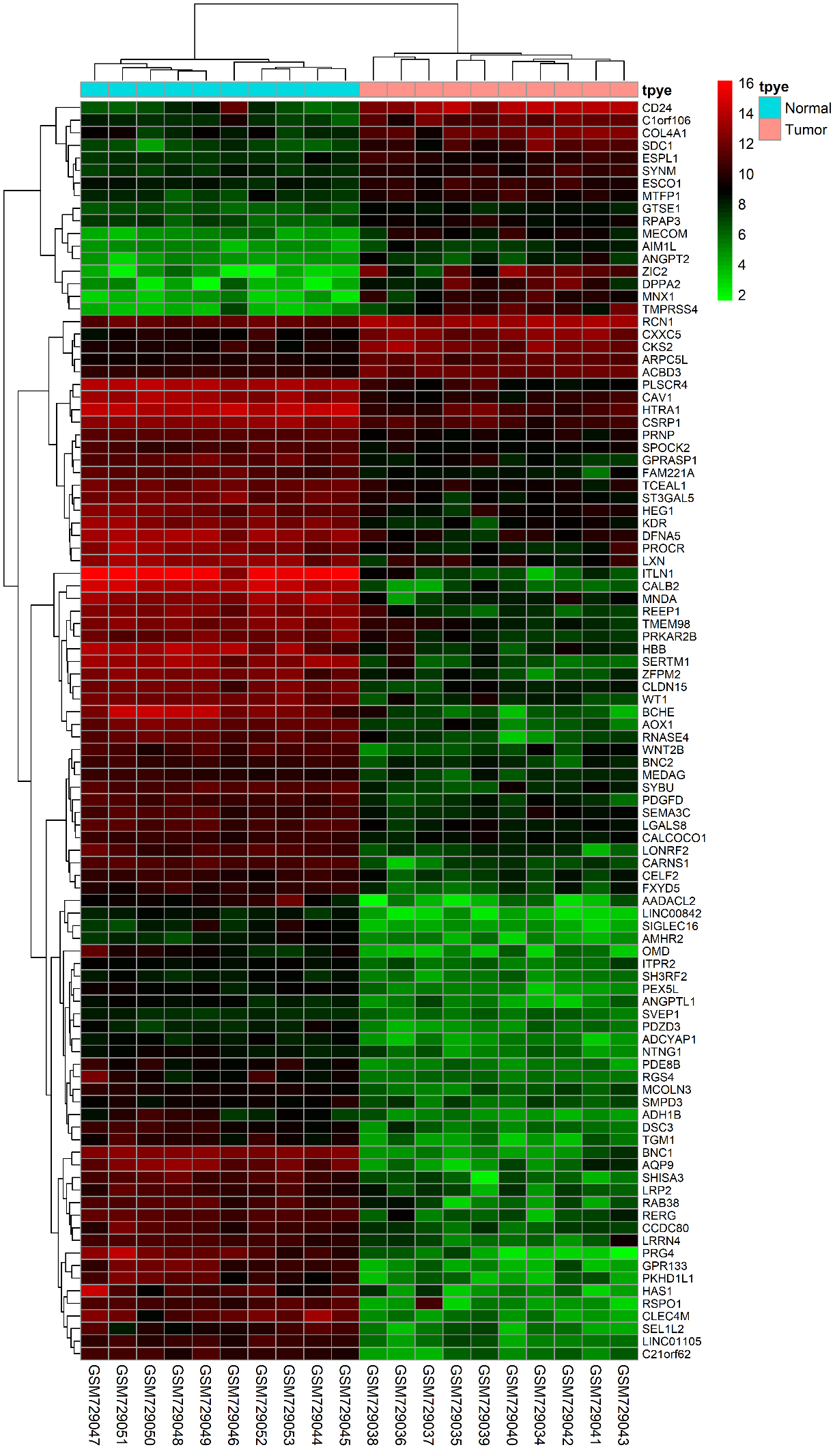


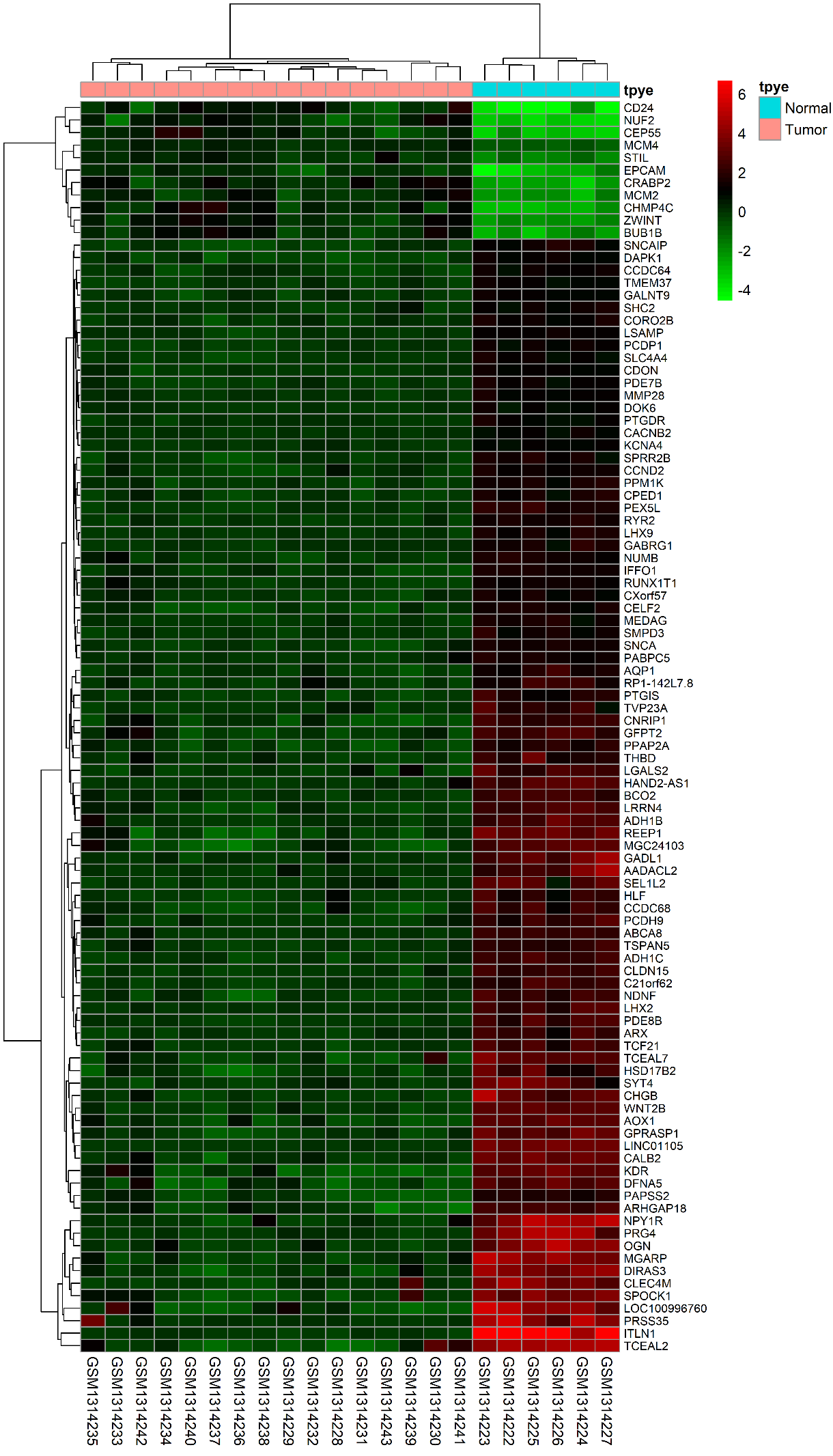


Supplement S1 Heatmap for GSE14407, GSE29450, and GSE54388.

Supplement: Supplementary file 1 — Additional file 1: Supplement S1 Heatmap for GSE14407, GSE29450, and GSE54388. [file 13048_2021_837_MOESM1_ESM.docx]
